# Predictors of employment in people with moderate to severe mental illness participating in a randomized controlled trial of Individual Placement and Support (IPS)

**DOI:** 10.1177/0020764020934841

**Published:** 2020-07-15

**Authors:** Tonje Fyhn, Simon Øverland, Silje E Reme

**Affiliations:** 1 NORCE, Bergen, Norway; 2 Norwegian Institute of Public Health, Bergen, Norway; 3 Department of Psychosocial Science, University of Bergen, Bergen, Norway; 4 Department of Psychology, University of Oslo, Oslo, Norway

**Keywords:** IPS, randomized controlled trial, mental illness, employment, predictors, involuntary hospitalization

## Abstract

**Background:**

Many people with moderate to severe mental illness have a desire to obtain ordinary employment. To aid further development of health and social services for this group, the aim of this study was to examine candidate modifiable and prognostic markers of employment, and moderating effects of group allocation in a clinical trial.

**Method:**

The sample consists of 327 patients in treatment for mental illness, randomized to Individual Placement and Support (IPS) or treatment as usual (TAU) as part of a clinical trial. Psychosocial and demographic baseline characteristics were included as predictors in log binary regression analyses with employment 18 months after inclusion as the outcome, and group allocation as the moderator (IPS or TAU).

**Results:**

Directive emotional support and non-directive instrumental support seemed to positively predict employment, but effects were small. Involuntary hospitalization seemed to be a strong negative predictor of employment. Group allocation did not moderate any main effects.

**Conclusion:**

Interpretation of the findings suggest that attention should be given to certain aspects of health and social services provided to this target group, and in particular the effect of receiving appropriate types of social support. The findings are novel because social support and involuntary hospitalization do not seem to have been included in previous predictor studies. The results from this study identify new topics for research on employment outcomes for this population.

## Background

Clinical practice in mental health care has gradually broadened its approach from strictly targeting symptoms, clinical status and expression, to enhancing recovery through participation and integration in society (Saxena et al., 2013; Slade, 2010). Employment is a major arena for the recovery process and for participation in society for adults, and there is substantial evidence that employment can improve functioning, finances and mental and general health (Bond et al., 2001; Drake & Whitley, 2014; Kukla et al., 2012; Rueda et al., 2012). Poor job quality, on the other hand, may deteriorate health (Welsh et al., 2016), even to the same level as being unemployed (Broom et al., 2006; Butterworth et al., 2011).

### Individual Placement and Support

The vocational rehabilitation service Individual Placement and Support (IPS) is a manual-based method within the Supported Employment (SE) paradigm, seeking to assist people with severe mental illness to obtain ordinary employment (Drake & Becker, 1996). IPS is based on eight principles: obtaining competitive employment, rapid job search, systematic job development, integrated services, benefits planning, zero exclusion, time-unlimited support and worker preferences (Becker & Drake, 2003). The method has proven consistently more effective than other vocational rehabilitation efforts and treatment as usual (TAU) for obtaining employment, across many different contexts (Frederick & VanderWeele, 2019). Although more effective than other vocational rehabilitation efforts, in many studies, the majority of participants receiving IPS remains unemployed at the time of follow-up (Heslin et al., 2011; Oshima et al., 2014; Reme et al., 2019; Suijkerbuijk et al., 2017; Viering et al., 2015). Knowledge about predictors across the individual, organizational and contextual domains might enhance the effect of IPS. This study aims to identify individual characteristics that may affect employment outcomes for patients with mental illness receiving IPS or usual care.

### Predictors of employment in previous studies

Research on predictors of employment for people with mental illness has mainly focused on demographic traits, illness variables and previous work experience. For demographic predictors, younger age (Campbell, 2007; Corbière et al., 2017; Wewiorski & Fabian, 2004) and higher education (Campbell, 2007; Cook et al., 2001; Nordt et al., 2007; Tse et al., 2014) are generally found to positively predict employment, although some studies have failed to establish these relationships (Catty et al., 2008; Corbière et al., 2011; Sanchez, 2018). Studies investigating illness variables (mainly symptom severity, diagnoses and hospitalizations) vary in their conclusions; however, symptom severity generally tends to be a negative predictor of employment (Biegel et al., 2010; Tse et al., 2014), while the effect of diagnoses vary from positive in a few studies (Cook et al., 2001; Nordt et al., 2007), to negative (Biegel et al., 2010), to no association in other studies (Campbell et al., 2010; Catty et al., 2008; Michon et al., 2005). Having been admitted to a psychiatric hospital seems to be the most consistent negative predictor of employment among commonly measured illness-related variables (Cook et al., 2001; Nordt et al., 2007; Tse et al., 2014). The effect of involuntarily hospitalization on employment outcomes does not seem to have been investigated in previous studies.

Social support is another variable not commonly included in studies investigating predictors of employment, although it has shown consistent positive associations with life outcomes such as positive health behavior, motivation and achievement (Cirik, 2015; Verheijden et al., 2005) and negative associations with morbidity and mortality (Cohen et al., 2000). Social support can refer to an individual’s social integration as well as the type or function of the support provided (Wills & Shinar, 2000). Fisher and colleagues (2004) distinguish between four types of social support measured along the dimensions directive/non-directive and emotional/instrumental which are included in this study. Satisfaction with the support usually depends on whether the type of support matches the recipient’s situation (Horowitz et al., 2001).

It is reasonable to assume that employment predictors will differ between a group of people with mental illness participating in an SE program and a general group of people with mental illness, given that the criteria for participation in IPS are based on participants’ expressed desire to obtain employment (Drake, 1998). Studies comparing predictors of employment between SE and general populations have identified more or less the same predictors; however, the associations seem to be weaker in the SE population (Campbell, 2007; McGurk & Mueser, 2004). One study analyzed data from four randomized controlled trials (RCTs) of IPS and found different predictors in the control group and the IPS group (Campbell et al., 2010). It is possible that part of the effectiveness of IPS occurs by enhancing the effect of positive predictors on employment outcomes and/or by mitigating the effects of negative predictors (Campbell et al., 2010; McGurk & Mueser, 2004). As the intervention is not based on psychological or intervention theories, not much is known about the underlying mechanisms facilitating its effects. Based on this, testing for effect modification of group allocation is a secondary aim in this study.

### Variables under study

The predictors in the study include age, level of education, four types of social support, symptom severity of depression and anxiety, and having been involuntarily committed to a psychiatric hospital. Involuntary hospitalization and social support do not seem to have been investigated for this purpose in previous studies, while the other variables have produced somewhat conflicting results. Investigating predictors of employment in this study population may increase the knowledge base of what traits or characteristics might need particular attention during follow-up. Moreover, moderating effects of group allocation (IPS vs. TAU) may also indicate whether IPS enhances the positive effect or ameliorates the negative effect of certain predictors. The aim of the study is therefore twofold: to investigate the effect of potential predictors on employment outcomes, as well as investigate whether group allocation moderates these effects. This may increase our understanding of modifiable predictors for employment in this population and give indications of how IPS works to help participants obtain employment.

## Materials and methods

The data material used in the study is from an RCT comparing TAU to IPS for individuals in treatment for moderate to severe mental illness (Reme et al., 2019). IPS was implemented in six centers across Norway. Inclusion started on 1 October 2013 and ended 31 October 2014. The study protocol (Sveinsdottir et al., 2014) and the outcome evaluation (Reme et al., 2019) are available elsewhere. The data used in this study include Mini-International Neuropsychiatric Interview (M.I.N.I.) psychiatric interviews conducted at inclusion, data from baseline questionnaires and register data on employment status 18 months after inclusion.

### Ethics and consent

The study was submitted to the Norwegian Regional Ethical Committee (REC; 28 May 2013; project no. 2013/960); however, since the main outcome of the study (employment) was not a health measure, it was not considered to fall under the Health Research Act (Ministry of Health and Care Services, 2008). The project was referred to the Norwegian Social Science Data Services, where permission was granted (4 October 2013; project no. 34989). Informed consent was signed by each participant in the study. The study complied with the Helsinki Declaration.

### Population

Inclusion criteria were that participants (a) were in treatment in psychiatric health care, (b) were not in employment but had a desire to obtain this and (c) had sufficient language skills to understand and respond to the questionnaires. The study population consists of 327 participants who at the time of inclusion were undergoing treatment for moderate to severe mental illness (184 randomized to the treatment group, 143 randomized to the control group). Originally, 408 participants were included in the trial (227 randomized to the treatment group, 181 randomized to the control group) (Reme et al., 2019). However, registry data and supplemental information obtained at a later point showed that 81 participants were either registered as employed at baseline or had obtained employment at 18 months through the use of wage subsidies. As the former violated the inclusion criteria of no employment, and the latter violated the IPS principle of ordinary employment, participants registered with employment at baseline were excluded from the study, while participants who obtained employment through wage subsidies were not treated as employed in the analyses (*n* = 9).

Mean age in the study population was 35 (*SD* = 10.72) years, and 50% were female. The M.I.N.I., a brief and structured screening interview (Sheehan et al., 1998), was conducted on 248 (76%) of the participants at inclusion to screen for psychiatric diagnoses. The intention was to conduct M.I.N.I. interviews with each participant through local staff, but this was not feasible due to various practical issues. There were no indications of systematic patterns in missing interviews. A total of 52% of participants were classified as having moderate mental illness (mainly anxiety), while 48% were classified as having severe mental illness (mainly psychotic disorders and severe depression).

### Treatment and control groups

The treatment group received IPS in addition to mental health treatment, and the control group received high-quality care as usual, in addition to mental health treatment. This implied being prioritized for a spot in a work-focused rehabilitation program offered by their local welfare office, such as ‘work with assistance’ or traineeship in an ordinary or sheltered business. All IPS centers participating in the study obtained fair or good fidelity during the study period according to the IPS Fidelity Scale (Fyhn et al., 2020; Kim et al., 2015).

### Recruitment and randomization

Participants were patients in secondary care, recruited from district psychiatric hospitals and local welfare administration offices. Potential participants were given thorough information about the study aim, what randomization means and why it is necessary to achieve the study aim, implications of participation and data protection. Eligible participants who wished to participate signed a consent form and filled out the baseline questionnaire. Participants were randomly allocated to the intervention or control group by a data-generated randomization list. The first 5 months the randomization ratio was 2:1 in favor of the intervention group to ensure that full capacity was reached at the IPS centers. When the inclusion period was over, 56% had been randomized to the intervention group, and 44% to the control group. Follow-up of the intervention group commenced subsequently. Control participants were referred to their caseworker at their local welfare office.

### Predictor variables

Predictor variables measured at baseline included age, education, social support, symptoms of anxiety and depression and whether one had experienced involuntary hospitalization.

#### Sociodemographic variables

The variables age and education level were measured through single items in the baseline questionnaire. Education was dummy-coded such that 0 = highest completed education was lower secondary (10 years) and 1 = highest completed education was high school or higher education.

#### Social support

Social support was measured by Fisher’s ‘Non-directive and directive support survey’ (Fisher et al., 2004). The 16-item version was used, which consists of the subscales non-directive instrumental support, directive instrumental support, directive emotional support and non-directive emotional support. Directive support is offered when the support provider assumes responsibility for tasks or choices on the behalf of the recipient (Stewart et al., 2012). Conversely, for non-directive support, the support provider seeks to cooperate with the recipient. Instrumental support offers practical assistance, while emotional support is directed at thoughts and feelings.

The respondent chooses one person in their life whom they complete the survey with reference to (e.g., doctor, family member, friend). Items are rated in reference to how typical the described behavior is for the reference person, on a scale from 1 = *not at all typical* to 5 = *very typical*. Example items are [the reference person] ‘Is available to talk anytime’ (non-directive emotional), ‘Pushes you to get going on things’ (directive emotional), ‘Takes charge of your problems’ (directive instrumental) and ‘Cooperates with you to get things done’ (non-directive instrumental).

#### Illness-related variables

Anxiety and depression symptoms were measured through the Hospital Anxiety and Depression Scale (HADS), which consists of seven items measuring depression symptoms and seven items measuring anxiety symptoms (Bjelland et al., 2002; Zigmond & Snaith, 1983). The responses are coded 0 to 3 according to the direction of the item. Examples of statements are ‘I feel tense or “wound up”’ (anxiety) and ‘I still enjoy the things I used to enjoy’ (depression). The sum score variable for HADS was used in the analysis.

Involuntary hospitalization (yes/no) was measured by the item ‘Have you ever been hospitalized involuntarily?’

Scores on predictor variables measured at baseline are presented in Table 1. For social support, approximately 40% of respondents referred to a health or social worker, while the rest were evenly distributed on the categories ‘partner’, ‘close family member’ or ‘other’.

**Table 1.** Baseline scores on variables under study.

Variable	*N*	%	*M*	*SD*
Age (*continuous*)	327		35.0	10.72
Lower secondary (10 years) as highest education level (*categorical*)	310	33		
Social support non-directive emotional, SSNE (*continuous*, 0–20)	300		16.0	3.56
Social support directive emotional, SSDE (*continuous*, 0–20)	300		12.2	3.72
Social support non-directive instrumental, SSNI (*continuous*, 0–20)	300		13.7	4.04
Social support directive instrumental, SSDI (*continuous*, 0–20)	300		12.3	4.19
Hospital Depression and Anxiety Scale (*continuous*, 0–42)	321		15.6	7.66
Involuntary hospitalization (*categorical*)	312	30		

No differences were found between the intervention and control groups at baseline.

### Outcome variable

The outcome variable was employment status at 18 months, as measured by the Norwegian Work and Welfare Administration’s (NAV) State Register of Employers and Employees (SREE). The variable was coded 1 for registered employment, and 0 for no registration of employment. The register is based on employers’ monthly registration of start and finish dates for their employees and includes all forms of employment assumed to exceed 1,000 NOK per year (app. 100 EUR). Jobs yielding smaller earnings than this are not registered as employment. The register provides an objective data source for the main outcome with no loss to follow-up, as compared to self-report. An 18-month time frame was chosen because the median length of follow-up for the IPS participants was 15 months, and because 18 months was assumed to be a more reliable indicator of sustainable workforce attachment than a shorter observation period.

### Statistical analyses

Main effects and effect modification were assessed in individual log binary regression analyses using SPSS 25. Continuous variables were centered and analyses were bootstrapped. Listwise deletion was used. Multi-collinearity was assessed prior to the analyses. Dichotomous variables were coded such that absence of a characteristic was coded 0 (reference category), and presence of a characteristic was coded 1.

## Results

Directive emotional and non-directive instrumental support seemed to positively predict employment, while involuntary hospitalization seemed to negatively predict employment. Results from the regression analyses are presented in Table 2.

**Table 2.** Results from binomial regression analyses.

Predictor variable	*B*	*SE*	χ^2^	RR	*p*	95% CI
Age (*continuous; n* = 327)	−0.03	0.02	2.6(1)	0.97	0.107	[0.94, 1.00]
Highest completed education (*categorical, n* = 310)^a^						
Upper secondary (13 years of school) or higher education	0.59	0.47	1.57(1)	1.18	0.210	[0.72, 4.49]
Social support non-directive emotional, SSNE (*continuous, n* = 300)	0.12	0.07	3.04(1)	1.13	0.081	[0.99, 1.30]
Social support directive emotional, SSDE (*continuous, n* = 300)	0.15	0.05	8.16(1)	1.16	0.004^**^	[1.05, 1.29]
Social support non-directive instrumental, SSNI (*continuous, n* = 300)	0.10	0.05	3.87(1)	1.11	0.049^*^	[1.00, 1.23]
Social support directive instrumental, SSDI (*continuous, n* = 300)	0.08	0.05	3.24(1)	1.09	0.072	[0.99, 1.19]
Hospital Anxiety and Depression Scale, HADS (*continuous, n* = 321)	0.31	0.02	1.71(1)	1.03	0.191	[0.99, 1.08]
Involuntary hospitalization (*categorical, n* = 312)	−1.48	0.72	4.3(1)	0.23^b^	0.038^*^	[0.06, 0.92]

Regression coefficient, standard error, Wald chi-square (*df*), risk ratio (RR), *p* value and 95% confidence interval for each predictor.

* p < 0.05 **p < 0.01

*SE*: standard error; CI: confidence interval.

a Reference category: Lower secondary. ^b^Inverted value.

The results indicate that for every unit increase on the social support scales, the odds of being employed at 18 months slightly increased; however, the effect sizes are very small, and interpretation should be made with caution. As for involuntary hospitalization, the results indicate that participants who had experienced this had 77% less likelihood of being employed at 18 months compared to those who had not been involuntarily hospitalized. None of the interaction terms included in the models were significant.

## Discussion

The aim of the study was to identify predictors of employment in a study population of patients with moderate to severe mental illness, who had an expressed desire to obtain ordinary employment. Furthermore, the aim was to identify whether group allocation modified these effects. The results indicate that directive emotional support and non-directive instrumental support positively predicted employment at 18 months follow-up, while having been involuntarily hospitalized was a negative predictor of employment. No moderating effects of group allocation could be detected.

Although the effects of social support on employment are small, it is worth noting that the two types of support standing out in the results are diametrically opposite. The findings suggest that directive emotional support, which is relational in nature, and non-directive instrumental support, which is practical in nature, may both benefit this target group in their search for employment. Previous studies of these different types of support suggest that non-directive support in particular has positive associations with various health-related behaviors, individual outcomes and workplace outcomes (Fisher et al., 1997, 2004; Stewart et al., 2012). Although directive support has shown detrimental effects on health and well-being, there are studies indicating that directive support can be beneficial for recipients in a vulnerable or acute situation (Fisher et al., 1997; Gabriele et al., 2011). The findings in this study seem to support this notion, as participants in the study were all in treatment for moderate to severe mental illness, and struggling with their workforce attachment. Studies in similar populations have found job search activities to be associated with obtaining employment (Corbière et al., 2011, 2017), and non-directive instrumental support may play a part in facilitating such activities, for example, through providing practical guidance on writing a CV, search strategies, the job interview and so on. It is noteworthy that the social support variables measure support provided by one specific person (such as a general practitioner or a family member), and is not a measure of general perceived support from one’s social network, or degree of social integration. This suggests that one trustworthy person in a patient’s life can have an impact on this outcome. Social support does not seem to have been previously studied as a predictor for obtaining employment, and the differentiation between different types of support should be included in future studies to increase our understanding of its function for this target group on several life outcomes. This can in turn support the development of purposeful social and health practices for this group, including understanding the role of significant others in a patient’s life.

Involuntary hospitalization seems to be a strong negative predictor of employment. Although it does not seem to have been included in previous studies, it does seem like hospitalizations with no specifications of voluntariness is a consistent negative predictor of employment (Cook et al., 2001; Nordt et al., 2007; Russinova et al., 2018). In this study, a multiple regression model examining possible confounding with anxiety and depression was tested, but was not significant, indicating that its association with employment is unique. There are no data on frequency or time passed since the involuntary hospitalization, meaning that its association with employment holds regardless of these variations. Studies exploring first-person accounts of involuntary hospitalizations find that patients express feelings of disempowerment, loss of autonomy, self-stigma and lack of involvement during commitment and treatment (Katsakou et al., 2012; Murphy et al., 2017; Nyttingnes et al., 2016; Rusch et al., 2014). The long-term effects of these experiences are unknown, but considering the current findings, it may be hypothesized that the experience increases self-stigma that negatively affect the search for employment, for example, through helplessness and low self-efficacy (Corrigan et al., 2009). Providers of vocational services might be able to identify and ameliorate the individual effects caused by involuntary hospitalization, for example, through a client-centered approach and active participation.

The other results of the study are less novel, yet important as they replicate findings in previous studies, and also support a broad approach to including this target group in work rehabilitation efforts by not excluding participants based on diagnoses. Symptoms of depression and anxiety, age and level of education did not predict employment in this study. Previous studies have generally found negative associations between illness symptoms and employment outcomes (Biegel et al., 2010; McGurk & Mueser, 2004; Tse et al., 2014), but findings in this study do not confirm this association. This could be due to power issues or it could simply be that these symptoms as measured through HADS do not have a negative effect on employment for this population. The lack of association between symptoms and employment in this study corresponds well with a recovery perspective, where illness in itself is not a barrier for seeking employment, as it does not necessarily hamper work functioning nor motivation to work (Anthony, 1993). The finding is also in line with the rationale behind the non-exclusion principle in IPS, stating that severity of illness or diagnosis is not a reason for exclusion from the program (Becker & Drake, 2003).

### Strengths and limitations

One strength of the study is that its outcome measure is based on register data rather than self-report, ensuring an objective data source with no loss to follow-up. The randomized controlled design enabled an examination of the moderating effect of group allocation on the association between predictors and employment outcomes. The study population is sufficiently large to generalize the main effects of the regression analyses to other populations of people with moderate to severe mental illness who are motivated to find ordinary employment. However, it was not large enough to provide sufficient power to the moderation analyses. Studies with larger subgroups might be able to detect differences in main effects between IPS and control groups should they exist and increase our knowledge about how IPS is effective in facilitating ordinary employment for people with moderate to severe mental illness. Another limitation is that the associations of the predictors with employment are bound up with the time frame of the investigation, which is 18 months from baseline. The results might differ for longer or shorter observation periods, which may be investigated in the planned follow-up studies of the same population. Although there is a risk of selection bias, the external validity of the trial was strengthened by the fact that it was a pragmatic trial studying an intervention under real-life conditions.

## Conclusion

The aim of the study was to investigate predictors of employment in a population diagnosed with and in treatment for moderate to severe mental illness and to investigate whether group allocation moderated these relationships. The results showed that directive emotional support and non-directive instrumental support seemed to positively predict employment status at 18 months, while involuntary hospitalization negatively predicted employment. None of these variables seem to have been studied as predictors of employment in this target group before. Age, education, symptom severity and non-directive emotional and directive instrumental support did not seem to be associated with employment outcomes. Group allocation did not moderate any main effects in this study. Future studies aiming to improve our understanding of effective health and social services for this target group should further explore the role of social support and involuntary hospitalization, to extend the findings in this study. As effect sizes for social support were small, these findings should be interpreted with caution, and future research should attempt to replicate and expand them.

## References

[bibr1-0020764020934841] Anthony W. A. (1993). Recovery from mental illness: The guiding vision of the mental health service system in the 1990s. Psychosocial Rehabilitation Journal, 16(4), 11–23.

[bibr2-0020764020934841] Becker D. R. Drake R. E. (2003). A working life for people with severe mental illness. Oxford University Press.

[bibr3-0020764020934841] Biegel D. E. Stevenson L. D. Beimers D. Ronis R. J. Boyle P. (2010). Predictors of competitive employment among consumers with co-occurring mental and substance use disorders. Research on Social Work Practice, 20(2), 191–201. 10.1177/1049731509333373

[bibr4-0020764020934841] Bjelland I. Dahl A. A. Haug T. T. Neckelmann D. (2002). The validity of the Hospital Anxiety and Depression Scale – An updated literature review. Journal of Psychosomatic Research, 52(2), 69–77. 10.1016/S0022-3999(01)00296-3 11832252

[bibr5-0020764020934841] Bond G. R. Resnick S. G. Drake R. E. Xie H. McHugo G. J. Bebout R. R. (2001). Does competitive employment improve nonvocational outcomes for people with severe mental illness? Journal of Consulting Clinical Psychology, 69(3), 489–501. 11495178 10.1037//0022-006x.69.3.489

[bibr6-0020764020934841] Broom D. H. D’Souza R. M. Strazdins L. Butterworth P. Parslow R. Rodgers B. (2006). The lesser evil: Bad jobs or unemployment? A survey of mid-aged Australians. Social Science & Medicine, 63(3), 575–586. 10.1016/j.socscimed.2006.02.003 16597477

[bibr7-0020764020934841] Butterworth P. Leach L. S. Strazdins L. Olesen S. C. Rodgers B. Broom D. H. (2011). The psychosocial quality of work determines whether employment has benefits for mental health: Results from a longitudinal national household panel survey. Occupational and Environmental Medicine, 68(11), 806–812. 21406384 10.1136/oem.2010.059030

[bibr8-0020764020934841] Campbell K. (2007). Consumer predictors of competitive employment outcomes in supported employment. Purdue University.

[bibr9-0020764020934841] Campbell K. Bond G. R. Drake R. E. McHugo G. J. Xie H. (2010). Client predictors of employment outcomes in high-fidelity supported employment: A regression analysis. Journal of Nervous and Mental Disease, 198(8), 556–563. 10.1097/NMD.0b013e3181ea1e53 20699720

[bibr10-0020764020934841] Catty J. Lissouba P. White S. Becker T. Drake R. E. Fioritti A. , . . . EQOLISE Group. (2008). Predictors of employment for people with severe mental illness: Results of an international six-centre randomised controlled trial. British Journal of Psychiatry, 192(3), 224–231. 10.1192/bjp.bp.107.041475 18310585

[bibr11-0020764020934841] Cirik I. (2015). Relationships between social support, motivation and science achievement: Structural equation modeling. Anthropologist, 20(2), 232–242.

[bibr12-0020764020934841] Cohen S. E. Underwood L. G. Gottlieb B. H. (2000). Social support measurement and intervention: A guide for health and social scientists. Oxford University Press.

[bibr13-0020764020934841] Cook J. A. Pickett-Schenk S. A. Grey D. Banghart M. Rosenheck F. Randolph F. (2001). Vocational outcomes among formerly homeless persons with severe mental illness in the ACCESS program. Psychiatric Services, 52(8), 1075–1080. 11474054 10.1176/appi.ps.52.8.1075

[bibr14-0020764020934841] Corbière M. Lecomte T. Reinharz D. Kirsh B. Goering P. Menear M. . . . Goldner E. M. (2017). Predictors of acquisition of competitive employment for people enrolled in supported employment programs. Journal of Nervous and Mental Disease, 205(4), 275–282. 10.1097/nmd.0000000000000612 28212170

[bibr15-0020764020934841] Corbière M. Zaniboni S. Lecomte T. Bond G. Gilles P. Lesage A. Goldner E. (2011). Job acquisition for people with severe mental illness enrolled in supported employment programs: A theoretically grounded empirical study. Journal of Occupational Rehabilitation, 21(3), 342–354. 21656251 10.1007/s10926-011-9315-3

[bibr16-0020764020934841] Corrigan P. W. Larson J. E. Rusch N. (2009). Self-stigma and the ‘why try’ effect: Impact on life goals and evidence-based practices. World Psychiatry, 8(2), 75–81. 19516923 10.1002/j.2051-5545.2009.tb00218.x PMC2694098

[bibr17-0020764020934841] Drake R. E. Becker D. R. (1996). The individual placement and support model of supported employment. Psychiatric Services, 47(5), 473–475. 8740486 10.1176/ps.47.5.473

[bibr18-0020764020934841] Drake. (1998). A brief history of the individual placement and support model. Psychiatric Rehabilitation Journal, 22(1), 3.

[bibr19-0020764020934841] Drake R. E. Whitley R. (2014). Recovery and severe mental illness: Description and analysis. The Canadian Journal of Psychiatry, 59(5), 236–242. 25007276 10.1177/070674371405900502 PMC4079142

[bibr20-0020764020934841] Fisher E. Everard K. Gabriele J. Heins J. Jeffe D. Scott C. Walker M. (2004). Measuring nondirective and directive social support. Division of Health Behavior Research, Washington University.

[bibr21-0020764020934841] Fisher E. B. LaGreca A. M. Greco P. Arfken C. Schneiderman N. (1997). Directive and nondirective social support in diabetes management. International Journal of Behavioral Medicine, 4(2), 131–144. 10.1207/s15327558ijbm0402_3 16250735

[bibr22-0020764020934841] Frederick D. E. VanderWeele T. J. (2019). Supported employment: Meta-analysis and review of randomized controlled trials of individual placement and support. PLOS ONE, 14(2), Article e0212208. 10.1371/journal.pone.0212208 PMC6382127 30785954

[bibr23-0020764020934841] Fyhn T. Ludvigsen K. Reme S. Schaafsma F. (2020). A structured process evaluation of a randomized controlled trial of Individual Placement and Support (IPS). Implementation Science Communications. 10.21203/rs.2.21755/v2 PMC7599092 33145494

[bibr24-0020764020934841] Gabriele J. M. Carpenter B. D. Tate D. F. Fisher E. B. (2011). Directive and nondirective e-coach support for weight loss in overweight adults. Annals of Behavioral Medicine, 41(2), 252–263. 10.1007/s12160-010-9240-2 21108032 PMC3891570

[bibr25-0020764020934841] Heslin M. Howard L. Leese M. McCrone P. Rice C. Jarrett M. . . . Thornicroft G. (2011). Randomized controlled trial of supported employment in England: 2 year follow-up of the Supported Work and Needs (SWAN) study. World Psychiatry, 10(2), 132–137. 21633690 10.1002/j.2051-5545.2011.tb00035.x PMC3105475

[bibr26-0020764020934841] Horowitz L. M. Krasnoperova E. N. Tatar D. G. Hansen M. B. Person E. A. Galvin K. L. Nelson K. L. (2001). The way to console may depend on the goal: Experimental studies of social support. Journal of Experimental Social Psychology, 37(1), 49–61. 10.1006/jesp.2000.1435

[bibr27-0020764020934841] Katsakou C. Rose D. Amos T. Bowers L. McCabe R. Oliver D. . . . Priebe S. (2012). Psychiatric patients’ views on why their involuntary hospitalisation was right or wrong: A qualitative study. Social Psychiatry and Psychiatric Epidemiology, 47(7), 1169–1179. 10.1007/s00127-011-0427-z 21863281

[bibr28-0020764020934841] Kim S. J. Bond D. R. Becker G. R. Swanson S. J. Langfitt-Reese S. (2015). Predictive validity of the Individual Placement and Support Fidelity Scale (IPS-25): A replication study. Journal of Vocational Rehabilitation, 43(3), 209–216.

[bibr29-0020764020934841] Kukla M. Bond G. R. Xie H. (2012). A prospective investigation of work and nonvocational outcomes in adults with severe mental illness. Journal of Nervous and Mental Disease, 200(3), 214–222. 10.1097/NMD.0b013e318247cb29 22373758

[bibr30-0020764020934841] McGurk S. R. Mueser K. T. (2004). Cognitive functioning, symptoms, and work in supported employment: A review and heuristic model. Schizophrenia Research, 70(2–3), 147–173. 10.1016/j.schres.2004.01.009 15329293

[bibr31-0020764020934841] Michon H. W. van Weeghel J. Kroon H. Schene A. H. (2005). Person-related predictors of employment outcomes after participation in psychiatric vocational rehabilitation programmes: A systematic review. Social Psychiatry and Psychiatric Epidemiology, 40(5), 408–416. 10.1007/s00127-005-0910-5 15902412

[bibr32-0020764020934841] Ministry of Health and Care Services. (2008). LOV-2008-06-20-44 §4a. The Act on Medical and Health Research (the Health Research Act). https://lovdata.no/dokument/NL/lov/2008-06-20-44

[bibr33-0020764020934841] Murphy R. McGuinness D. Bainbridge E. Brosnan L. Felzmann H. Keys M. . . . Higgins A. (2017). Service users’ experiences of involuntary hospital admission under the Mental Health Act 2001 in the Republic of Ireland. Psychiatric Services, 68(11), 1127–1135. 10.1176/appi.ps.201700008 28669292

[bibr34-0020764020934841] Nordt C. Muller B. Rossler W. Lauber C. (2007). Predictors and course of vocational status income, and quality of life in people with severe mental illness: A naturalistic study. Social Science & Medicine, 65(7), 1420–1429. 10.1016/j.socscimed.2007.05.024 17583402

[bibr35-0020764020934841] Nyttingnes O. Ruud T. Rugkasa J. (2016). ‘It’s unbelievably humiliating’ – Patients’ expressions of negative effects of coercion in mental health care. International Journal of Law and Psychiatry, 49, 147–153. 10.1016/j.ijlp.2016.08.009 27726890

[bibr36-0020764020934841] Oshima I. Sono T. Bond G. R. Nishio M. Ito J. (2014). A randomized controlled trial of individual placement and support in Japan. Psychiatric Rehabilitation Journal, 37(2), 137–143. 24912063 10.1037/prj0000085

[bibr37-0020764020934841] Reme S. E. Monstad K. Fyhn T. Sveinsdottir V. Løvvik C. Lie S. A. Øverland S. (2019). A randomized controlled multicenter trial of individual placement and support for patients with moderate-to-severe mental illness. Scandinavian Journal of Work, Environment & Health, 45, 33–41. 10.5271/sjweh.3753 30074050

[bibr38-0020764020934841] Rueda S. Chambers L. Wilson M. Mustard C. Rourke S. B. Bayoumi A. . . . Lavis J. (2012). Association of returning to work with better health in working-aged adults: A systematic review. American Journal of Public Health, 102(3), 541–556. 10.2105/Ajph.2011.300401 22390520 PMC3487667

[bibr39-0020764020934841] Rusch N. Muller M. Lay B. Corrigan P. W. Zahn T. Schonenberger M. . . . Rossler W. (2014). Emotional reactions to involuntary psychiatric hospitalization and stigma-related stress among people with mental illness. European Archives of Psychiatry and Clinical Neuroscience, 264(1), 35–43. 10.1007/s00406-013-0412-5 23689838

[bibr40-0020764020934841] Russinova Z. Bloch P. Wewiorski N. Shappell H. Rogers E. S. (2018). Predictors of sustained employment among individuals with serious mental illness: Findings from a 5-year naturalistic longitudinal study. Journal of Nervous and Mental Disease, 206(9), 669–679. 10.1097/nmd.0000000000000876 30124576

[bibr41-0020764020934841] Sanchez J. (2018). Employment predictors and outcomes of US state-federal vocational rehabilitation consumers with affective disorders: A CHAID analysis. Journal of Affective Disorders, 239, 48–57. 10.1016/j.jad.2018.06.044 29990662

[bibr42-0020764020934841] Saxena S. Funk M. Chisholm D. (2013). World health assembly adopts comprehensive mental health action plan 2013–2020. The Lancet, 381(9882), 1970–1971. 10.1016/S0140-6736(13)61139-3 23746771

[bibr43-0020764020934841] Sheehan D. V. Lecrubier Y. Sheehan K. H. Amorim P. Janavs J. Weiller E. . . . Dunbar G. (1998). The Mini-International Neuropsychiatric Interview (MINI): The development and validation of a structured diagnostic psychiatric interview for DSM-IV and ICD-10. The Journal of Clinical Psychiatry, 59, 22–33. 9881538

[bibr44-0020764020934841] Slade M. (2010). Mental illness and well-being: The central importance of positive psychology and recovery approaches. BMC Health Services Research, 10(1), Article 26. 20102609 10.1186/1472-6963-10-26 PMC2835700

[bibr45-0020764020934841] Stewart D. W. Gabriele J. M. Fisher E. B. (2012). Directive support, nondirective support, and health behaviors in a community sample. Journal of Behavioral Medicine, 35(5), 492–499. 10.1007/s10865-011-9377-x 21877174

[bibr46-0020764020934841] Suijkerbuijk T. B. Schaafsma F. G. van Mechelen J. C. Ojajarvi A. Corbiere M. Anema J. R. (2017). Interventions for obtaining and maintaining employment in adults with severe mental illness: A network meta-analysis. Cochrane Database of Systematic Reviews, 9, Article CD011867. 10.1002/14651858.CD011867.pub2 PMC6483771 28898402

[bibr47-0020764020934841] Sveinsdottir V. Løvvik C. Fyhn T. Monstad K. Ludvigsen K. Øverland S. Reme S. E. (2014). Protocol for the effect evaluation of Individual Placement and Support (IPS): A randomized controlled multicenter trial of IPS versus treatment as usual for patients with moderate to severe mental illness in Norway. BMC Psychiatry, 14(1), Article 307. 25403470 10.1186/s12888-014-0307-7 PMC4244061

[bibr48-0020764020934841] Tse S. Chan S. Ng K. L. Yatham L. N. (2014). Meta-analysis of predictors of favorable employment outcomes among individuals with bipolar disorder. Bipolar Disorders, 16(3), 217–229. 10.1111/bdi.12148 24219657

[bibr49-0020764020934841] Verheijden M. W. Bakx J. C. van Weel C. Koelen M. A. van Staveren W. A. (2005). Role of social support in lifestyle-focused weight management interventions. European Journal of Clinical Nutrition, 59(1), S179–S186. 10.1038/sj.ejcn.1602194 16052189

[bibr50-0020764020934841] Viering S. Jäger M. Bärtsch B. Nordt C. Rössler W. Warnke I. Kawohl W. (2015). Supported Employment for the reintegration of disability pensioners with mental illnesses: A randomized controlled trial. Frontiers in Public Health, 3, Article 237. 10.3389/fpubh.2015.00237 PMC4611964 26539425

[bibr51-0020764020934841] Welsh J. Strazdins L. Charlesworth S. Kulik C. T. Butterworth P. (2016). Health or harm? A cohort study of the importance of job quality in extended workforce participation by older adults. BMC Public Health, 16(1), Article 885. 27561448 10.1186/s12889-016-3478-y PMC5000457

[bibr52-0020764020934841] Wewiorski N. J. Fabian E. S. (2004). Association between demographic and diagnostic factors and employment outcomes for people with psychiatric disabilities: A synthesis of recent research. Mental Health Services Research, 6(1), 9–21. 15002677 10.1023/b:mhsr.0000011253.36712.15

[bibr53-0020764020934841] Wills T. A. Shinar O. (2000). Measuring perceived and received social support. In Cohen S. Underwood L. G. Gottlieb B. H. (Eds.), Social support measurement and intervention: A guide for health and social scientists (pp. 86–135). Oxford University Press.

[bibr54-0020764020934841] Zigmond A. S. Snaith R. P. (1983). The Hospital Anxiety and Depression Scale. Acta Psychiatrica Scandinavica, 67(6), 361–370. 6880820 10.1111/j.1600-0447.1983.tb09716.x

